# 6-Methyl-2,7-diphenyl-1,4-diazepan-5-one

**DOI:** 10.1107/S1600536809049630

**Published:** 2009-11-25

**Authors:** S. Sathish Kumar, Helen P. Kavitha, Jasmine P. Vennila, G. Chakkaravarthi, V. Manivannan

**Affiliations:** a Department of Chemistry, SRM University, Ramapuram Campus, Chennai 600 089, India; b Department of Physics, Panimalar Institute of Technology, Chennai 602103, Tamil Nadu, India; c Department of Physics, CPCL Polytechnic College, Chennai 600 068, India; d Department of Research and Development, PRIST University, Vallam, Thanjavur 613 403, Tamil Nadu, India

## Abstract

The title compound, C_18_H_20_N_2_O, crystallizes with two mol­ecules in the asymmetric unit. The seven-membered ring in both mol­ecules adopts a distorted chair conformation. The dihedral angles between the phenyl rings are 43.2 (1) and 54.7 (1)° in the two mol­ecules. The crystal packing features N—H⋯O and weak N—H⋯π and C—H⋯π inter­actions.

## Related literature

For the biological activity of related compounds, see: Gopalakrishnan *et al.* (2007 ▶); Wlodarczyk *et al.* (2006 ▶). For the synthetic procedure, see: Thennarasu & Perumal (2002 ▶). For hydrogen-bond motifs, see: Bernstein *et al.* (1995 ▶).
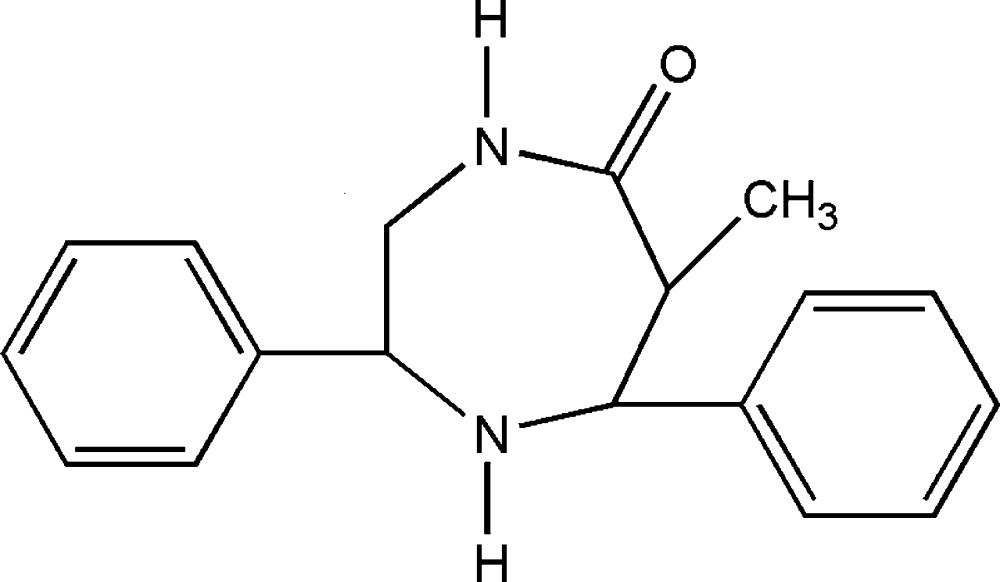


## Experimental

### 

#### Crystal data

C_18_H_20_N_2_O
*M* *_r_* = 280.36
Monoclinic, 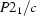

*a* = 10.8621 (3) Å
*b* = 21.3210 (7) Å
*c* = 13.3890 (4) Å
β = 91.167 (2)°
*V* = 3100.13 (16) Å^3^
*Z* = 8
Mo *K*α radiation
μ = 0.08 mm^−1^
*T* = 295 K
0.26 × 0.22 × 0.18 mm

#### Data collection

Bruker Kappa APEXII diffractometer
Absorption correction: multi-scan (*SADABS*; Sheldrick, 1996 ▶) *T*_min_ = 0.981, *T*_max_ = 0.987
32883 measured reflections
7003 independent reflections
4175 reflections with *I* > 2σ(*I*)
*R*_int_ = 0.034

#### Refinement

*R*[*F* ^2^ > 2σ(*F* ^2^)] = 0.049
*wR*(*F* ^2^) = 0.150
*S* = 1.01
7003 reflections
397 parameters
1 restraint
H atoms treated by a mixture of independent and constrained refinement
Δρ_max_ = 0.26 e Å^−3^
Δρ_min_ = −0.18 e Å^−3^

### 

Data collection: *APEX2* (Bruker, 2004 ▶); cell refinement: *SAINT* (Bruker, 2004 ▶); data reduction: *SAINT*; program(s) used to solve structure: *SHELXS97* (Sheldrick, 2008 ▶); program(s) used to refine structure: *SHELXL97* (Sheldrick, 2008 ▶); molecular graphics: *PLATON* (Spek, 2009 ▶); software used to prepare material for publication: *SHELXL97*.

## Supplementary Material

Crystal structure: contains datablocks global, I. DOI: 10.1107/S1600536809049630/gk2234sup1.cif

Structure factors: contains datablocks I. DOI: 10.1107/S1600536809049630/gk2234Isup2.hkl

Additional supplementary materials:  crystallographic information; 3D view; checkCIF report

## Figures and Tables

**Table 1:** Hydrogen-bond geometry (Å, °)

*D*—H⋯*A*	*D*—H	H⋯*A*	*D*⋯*A*	*D*—H⋯*A*
N2—H2*A*⋯O2^i^	0.90 (2)	2.02 (2)	2.911 (2)	170.2 (18)
N4—H4*A*⋯O1^i^	0.87 (2)	2.03 (2)	2.884 (2)	167 (2)
N1—H1*A*⋯*Cg*1^ii^	0.88 (2)	2.93 (2)	3.707 (2)	149.5 (2)
C18—H18⋯*Cg*5^iii^	0.93	2.95	3.872 (2)	171

Symmetry codes: (i) 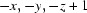; (ii) 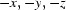; (iii) 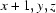. *Cg*1 and *Cg*5 are the centroids of the C1–C6 and C31–C36 rings, respectively.

**Fig. 1.**
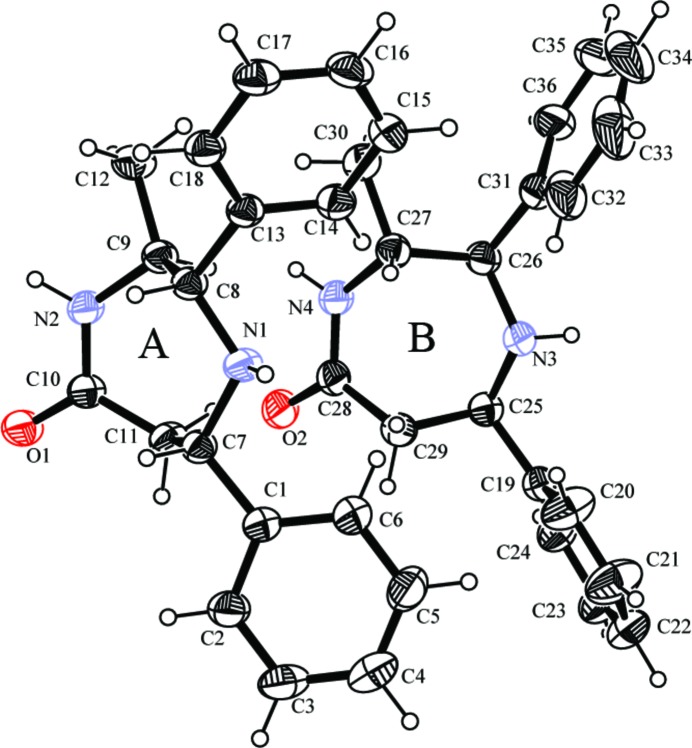
The molecular structure of the title compound, with atom labels and 30% probability displacement ellipsoids for non-H atoms. A and B are the two molecules in the asymmetric unit.

**Fig. 2.**
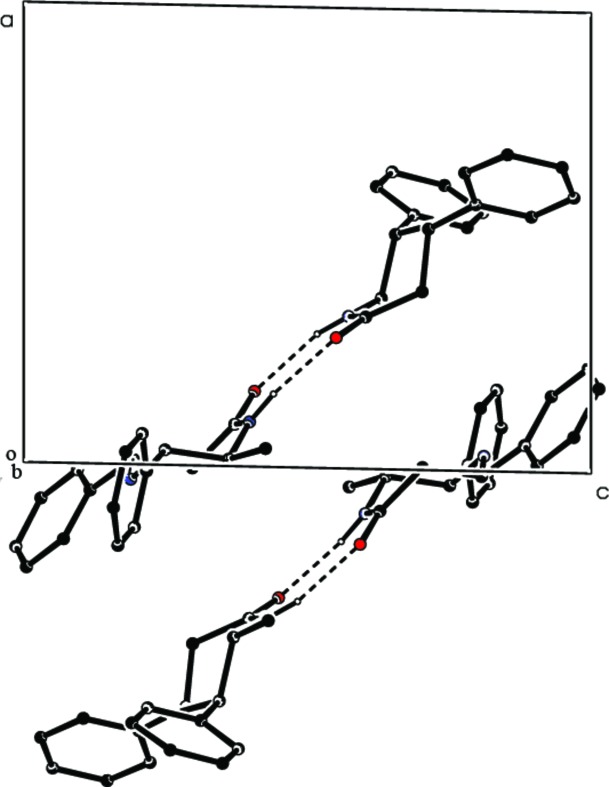
Intermolecular N—H···O interactions - view down the b axis. Hydrogen bonds are shown as dashed lines. H atoms not involved in hydrogen bonding have been omitted.

## supplementary crystallographic information

### Comment

In view of the biological activities of the heterocyclic compounds with 1,4-diazepan-5-one fragment that have widespread applications in pharmaceuticals (Wlodarczyk *et al.*, 2006; Gopalakrishnan *et al.*, 2007), we report the crystal structure of the title compound.

In the molecule A atoms C7, C8 and C9 show S-configuration whereas in the enatiomeric molecule B atoms C25, C26 and C27 show *R*-configuration. The phenyl ring C1—C6 forms the dihedral angle of 43.2 (1)° with the phenyl ring C13—C18 in molecule A and the phenyl ring C19—C24 forms the dihedral angle of 54.7 (1) ° with the phenyl ring C31—C36 in molecule B. Intermolecular N—H··· O interactions between the two symmetry independent molecules generates an eight-membered ring with graph-set motif *R*_2_^2^(8) (Bernstein *et al.*, 1995). The crystal packing is controlled by weak N—H··· O, N—H···π and C—H···π interactions (see Table 1 & Fig. 2).

### Experimental

The title compound was prepared according to the general procedure reported by Thennarasu & Perumal (2002). 3-Methyl-2,6-diphenylpiperidin-4-one (1.82 g, 5 mmole) was added into the ice cold sulfuric acid and the mixture was allowed to reach the room temperature. Then, sodium azide (1.92 g, 30 mmole) was added in portions over a period of one hour. The solution was then poured into crushed ice. The pH of the solution was adjusted to approximately 8.0 using 2 N NaOH solution. The precipitated white solid was recrystallized from ethanol to yield colourless diffraction quality crystals.

### Refinement

The H atoms from the N—H groups were located in difference Fourier maps and their positions and isotropic displacement parameters were freely refined. All other H atoms were positioned geometrically and refined using riding model with C—H = 0.93 Å and *U*_iso_(H) = 1.2U_eq_(C) for aromatic C—H, C—H = 0.98 Å and *U*_iso_(H) = 1.2U_eq_(C) for methine C—H, C—H = 0.97 Å and *U*_iso_(H) = 1.2U_eq_(C) for methylene C—H and C—H = 0.96 Å and *U*_iso_(H) = 1.5U_eq_(C) for methyl. The components of the anisotropic displacement parameters in direction of the bond of C33 and C34 were restrained to be equal within an effective standard deviation of 0.001 using the DELU command in *SHELXL97* (Sheldrick, 2008).

### Crystal data

**Table:**

C_18_H_20_N_2_O	*F*(000) = 1200
*M**_r_* = 280.36	*D*_x_ = 1.201 Mg m^−^^3^
Monoclinic, *P*2_1_/*c*	Mo *K*α radiation, λ = 0.71073 Å
Hall symbol: -P 2ybc	Cell parameters from 2786 reflections
*a* = 10.8621 (3) Å	θ = 1.9–27.4°
*b* = 21.3210 (7) Å	µ = 0.08 mm^−^^1^
*c* = 13.3890 (4) Å	*T* = 295 K
β = 91.167 (2)°	Block, colourless
*V* = 3100.13 (16) Å^3^	0.26 × 0.22 × 0.18 mm
*Z* = 8	

### Data collection

**Table:**

Bruker Kappa APEXII diffractometer	7003 independent reflections
Radiation source: fine-focus sealed tube	4175 reflections with *I* > 2σ(*I*)
graphite	*R*_int_ = 0.034
ω and φ scans	θ_max_ = 27.4°, θ_min_ = 1.9°
Absorption correction: multi-scan (*SADABS*; Sheldrick, 1996)	*h* = −14→13
*T*_min_ = 0.981, *T*_max_ = 0.987	*k* = −27→27
32883 measured reflections	*l* = −17→17

### Refinement

**Table:**

Refinement on *F*^2^	Primary atom site location: structure-invariant direct methods
Least-squares matrix: full	Secondary atom site location: difference Fourier map
*R*[*F*^2^ > 2σ(*F*^2^)] = 0.049	Hydrogen site location: inferred from neighbouring sites
*wR*(*F*^2^) = 0.150	H atoms treated by a mixture of independent and constrained refinement
*S* = 1.01	*w* = 1/[σ^2^(*F*_o_^2^) + (0.0613*P*)^2^ + 0.757*P*] where *P* = (*F*_o_^2^ + 2*F*_c_^2^)/3
7003 reflections	(Δ/σ)_max_ < 0.001
397 parameters	Δρ_max_ = 0.26 e Å^−^^3^
1 restraint	Δρ_min_ = −0.18 e Å^−^^3^

### Fractional atomic coordinates and isotropic or equivalent isotropic displacement parameters (Å^2^)

**Table:**

	*x*	*y*	*z*	*U*_iso_*/*U*_eq_	
C1	−0.05795 (17)	−0.04801 (8)	0.11713 (12)	0.0485 (4)	
C2	−0.0143 (2)	−0.10676 (9)	0.09516 (15)	0.0678 (6)	
H2	0.0593	−0.1202	0.1245	0.081*	
C3	−0.0773 (3)	−0.14624 (10)	0.03052 (16)	0.0795 (7)	
H3	−0.0463	−0.1860	0.0173	0.095*	
C4	−0.1837 (2)	−0.12746 (12)	−0.01365 (17)	0.0785 (7)	
H4	−0.2269	−0.1543	−0.0565	0.094*	
C5	−0.2275 (2)	−0.06876 (13)	0.00506 (18)	0.0847 (7)	
H5	−0.2998	−0.0552	−0.0264	0.102*	
C6	−0.16502 (19)	−0.02932 (11)	0.07031 (16)	0.0687 (6)	
H6	−0.1961	0.0105	0.0826	0.082*	
C7	0.01134 (17)	−0.00817 (8)	0.19307 (12)	0.0473 (4)	
H7	0.0991	−0.0087	0.1775	0.057*	
C8	0.03251 (17)	0.10337 (7)	0.24911 (12)	0.0463 (4)	
H8	0.1206	0.1007	0.2351	0.056*	
C9	0.01501 (17)	0.09158 (8)	0.36136 (12)	0.0493 (4)	
H9	−0.0702	0.0782	0.3712	0.059*	
C10	0.09125 (18)	−0.01783 (8)	0.37215 (13)	0.0526 (4)	
C11	−0.00555 (19)	−0.03663 (8)	0.29722 (13)	0.0549 (5)	
H11A	−0.0853	−0.0241	0.3216	0.066*	
H11B	−0.0056	−0.0820	0.2914	0.066*	
C12	0.0399 (2)	0.14835 (9)	0.42625 (14)	0.0662 (6)	
H12A	0.0341	0.1368	0.4953	0.099*	
H12B	−0.0197	0.1804	0.4108	0.099*	
H12C	0.1210	0.1640	0.4139	0.099*	
C13	−0.01396 (17)	0.16719 (8)	0.21681 (12)	0.0476 (4)	
C14	−0.13663 (18)	0.17781 (9)	0.19508 (14)	0.0562 (5)	
H14	−0.1928	0.1451	0.1998	0.067*	
C15	−0.1770 (2)	0.23685 (9)	0.16622 (15)	0.0643 (5)	
H15	−0.2601	0.2436	0.1522	0.077*	
C16	−0.0951 (2)	0.28531 (9)	0.15823 (15)	0.0663 (6)	
H16	−0.1222	0.3248	0.1383	0.080*	
C17	0.0266 (2)	0.27512 (9)	0.17977 (15)	0.0637 (5)	
H17	0.0825	0.3079	0.1747	0.076*	
C18	0.06729 (18)	0.21665 (8)	0.20889 (14)	0.0556 (5)	
H18	0.1505	0.2104	0.2234	0.067*	
C19	−0.57859 (17)	−0.06524 (9)	0.20328 (13)	0.0528 (4)	
C20	−0.5541 (3)	−0.05228 (12)	0.10529 (16)	0.0890 (8)	
H20	−0.5076	−0.0171	0.0896	0.107*	
C21	−0.5974 (3)	−0.09052 (13)	0.03036 (18)	0.1049 (9)	
H21	−0.5814	−0.0806	−0.0357	0.126*	
C22	−0.6634 (2)	−0.14265 (12)	0.05125 (19)	0.0848 (7)	
H22	−0.6910	−0.1690	0.0001	0.102*	
C23	−0.6884 (2)	−0.15582 (11)	0.14736 (19)	0.0822 (7)	
H23	−0.7340	−0.1914	0.1625	0.099*	
C24	−0.6472 (2)	−0.11726 (10)	0.22268 (16)	0.0685 (6)	
H24	−0.6664	−0.1267	0.2884	0.082*	
C25	−0.52358 (17)	−0.02607 (8)	0.28651 (13)	0.0519 (4)	
H25	−0.5652	−0.0359	0.3489	0.062*	
C26	−0.51055 (17)	0.08491 (8)	0.34472 (14)	0.0545 (5)	
H26	−0.5551	0.0724	0.4044	0.065*	
C27	−0.37168 (17)	0.08639 (8)	0.36986 (14)	0.0534 (4)	
H27	−0.3269	0.0873	0.3071	0.064*	
C28	−0.32986 (17)	−0.02759 (9)	0.39725 (14)	0.0557 (5)	
C29	−0.38718 (18)	−0.04257 (9)	0.29788 (14)	0.0602 (5)	
H29A	−0.3775	−0.0871	0.2855	0.072*	
H29B	−0.3424	−0.0203	0.2469	0.072*	
C30	−0.3340 (2)	0.14317 (10)	0.43108 (18)	0.0744 (6)	
H30A	−0.3845	0.1460	0.4889	0.112*	
H30B	−0.3442	0.1804	0.3914	0.112*	
H30C	−0.2492	0.1392	0.4518	0.112*	
C31	−0.55368 (18)	0.14851 (10)	0.31237 (19)	0.0691 (6)	
C32	−0.5286 (2)	0.17105 (11)	0.2185 (2)	0.0944 (8)	
H32	−0.4888	0.1454	0.1731	0.113*	
C33	−0.5619 (3)	0.23130 (17)	0.1909 (4)	0.1510 (17)	
H33	−0.5456	0.2459	0.1271	0.181*	
C34	−0.6192 (4)	0.26937 (18)	0.2584 (6)	0.180 (3)	
H34	−0.6395	0.3104	0.2413	0.216*	
C35	−0.6460 (3)	0.24657 (18)	0.3505 (5)	0.161 (2)	
H35	−0.6870	0.2720	0.3954	0.193*	
C36	−0.6138 (2)	0.18703 (12)	0.3781 (3)	0.1021 (10)	
H36	−0.6324	0.1724	0.4414	0.122*	
N1	−0.03260 (15)	0.05628 (7)	0.18881 (11)	0.0518 (4)	
N2	0.09714 (16)	0.04195 (7)	0.39814 (11)	0.0550 (4)	
N3	−0.53901 (17)	0.04046 (7)	0.26410 (12)	0.0566 (4)	
N4	−0.33046 (16)	0.03138 (8)	0.42746 (12)	0.0571 (4)	
O1	0.16394 (14)	−0.05709 (6)	0.40701 (10)	0.0724 (4)	
O2	−0.28387 (14)	−0.06962 (6)	0.44918 (11)	0.0751 (4)	
H1A	−0.0273 (17)	0.0686 (9)	0.1266 (15)	0.060 (6)*	
H2A	0.1588 (18)	0.0538 (9)	0.4403 (15)	0.065 (6)*	
H3A	−0.616 (2)	0.0465 (10)	0.2477 (16)	0.077 (7)*	
H4A	−0.291 (2)	0.0403 (10)	0.4827 (17)	0.073 (7)*	

### Atomic displacement parameters (Å^2^)

**Table:**

	*U*^11^	*U*^22^	*U*^33^	*U*^12^	*U*^13^	*U*^23^
C1	0.0583 (11)	0.0441 (10)	0.0432 (9)	−0.0040 (8)	0.0021 (8)	0.0013 (7)
C2	0.0880 (16)	0.0517 (12)	0.0632 (12)	0.0099 (10)	−0.0133 (11)	−0.0067 (9)
C3	0.120 (2)	0.0524 (13)	0.0659 (13)	−0.0023 (13)	−0.0062 (13)	−0.0130 (10)
C4	0.0900 (17)	0.0830 (17)	0.0627 (13)	−0.0259 (14)	0.0033 (12)	−0.0208 (12)
C5	0.0633 (14)	0.111 (2)	0.0794 (15)	−0.0023 (13)	−0.0112 (11)	−0.0298 (14)
C6	0.0635 (13)	0.0708 (14)	0.0713 (13)	0.0067 (10)	−0.0083 (10)	−0.0169 (11)
C7	0.0540 (10)	0.0389 (9)	0.0487 (9)	−0.0007 (8)	−0.0040 (8)	0.0017 (7)
C8	0.0548 (11)	0.0380 (9)	0.0460 (9)	0.0003 (7)	−0.0025 (8)	0.0015 (7)
C9	0.0594 (11)	0.0429 (10)	0.0454 (9)	0.0030 (8)	−0.0038 (8)	0.0003 (7)
C10	0.0696 (12)	0.0448 (10)	0.0430 (9)	0.0034 (9)	−0.0063 (8)	0.0032 (8)
C11	0.0725 (13)	0.0401 (10)	0.0518 (10)	−0.0040 (9)	−0.0083 (9)	0.0048 (8)
C12	0.0933 (16)	0.0515 (11)	0.0533 (11)	0.0069 (10)	−0.0063 (10)	−0.0058 (9)
C13	0.0605 (11)	0.0397 (9)	0.0425 (9)	0.0014 (8)	−0.0036 (8)	0.0000 (7)
C14	0.0619 (12)	0.0448 (10)	0.0618 (11)	−0.0033 (9)	−0.0036 (9)	0.0008 (8)
C15	0.0677 (13)	0.0550 (12)	0.0697 (13)	0.0111 (10)	−0.0122 (10)	−0.0024 (10)
C16	0.0933 (17)	0.0392 (11)	0.0660 (12)	0.0083 (10)	−0.0054 (11)	0.0028 (9)
C17	0.0812 (15)	0.0404 (10)	0.0696 (12)	−0.0061 (10)	0.0014 (11)	−0.0004 (9)
C18	0.0632 (12)	0.0445 (10)	0.0588 (11)	−0.0032 (9)	−0.0033 (9)	−0.0008 (8)
C19	0.0559 (11)	0.0503 (11)	0.0522 (10)	0.0020 (8)	−0.0043 (8)	0.0017 (8)
C20	0.136 (2)	0.0767 (16)	0.0541 (12)	−0.0326 (15)	0.0007 (13)	0.0039 (11)
C21	0.165 (3)	0.096 (2)	0.0536 (13)	−0.027 (2)	−0.0054 (15)	−0.0060 (13)
C22	0.0978 (18)	0.0840 (17)	0.0718 (16)	−0.0069 (14)	−0.0201 (13)	−0.0205 (13)
C23	0.0805 (16)	0.0777 (16)	0.0879 (17)	−0.0227 (13)	−0.0094 (13)	−0.0082 (13)
C24	0.0713 (14)	0.0710 (14)	0.0632 (12)	−0.0150 (11)	−0.0001 (10)	0.0004 (11)
C25	0.0588 (11)	0.0487 (10)	0.0483 (9)	−0.0006 (8)	0.0012 (8)	0.0039 (8)
C26	0.0548 (11)	0.0483 (10)	0.0605 (11)	0.0006 (8)	0.0019 (9)	−0.0014 (9)
C27	0.0543 (11)	0.0501 (11)	0.0557 (10)	0.0002 (8)	0.0000 (8)	0.0034 (8)
C28	0.0569 (12)	0.0542 (11)	0.0556 (10)	0.0029 (9)	−0.0049 (9)	0.0044 (9)
C29	0.0682 (13)	0.0526 (11)	0.0596 (11)	0.0076 (9)	−0.0064 (9)	−0.0045 (9)
C30	0.0751 (15)	0.0560 (13)	0.0912 (16)	−0.0024 (10)	−0.0197 (12)	−0.0040 (11)
C31	0.0543 (12)	0.0509 (12)	0.1012 (17)	0.0008 (9)	−0.0175 (11)	−0.0044 (12)
C32	0.0843 (17)	0.0668 (15)	0.131 (2)	−0.0035 (12)	−0.0251 (16)	0.0311 (15)
C33	0.105 (3)	0.083 (2)	0.263 (5)	−0.0263 (18)	−0.072 (3)	0.079 (3)
C34	0.096 (3)	0.0458 (19)	0.395 (8)	−0.0086 (17)	−0.079 (4)	0.031 (3)
C35	0.084 (2)	0.067 (2)	0.331 (7)	0.0177 (19)	−0.044 (3)	−0.062 (3)
C36	0.0637 (15)	0.0716 (16)	0.170 (3)	0.0132 (12)	−0.0193 (16)	−0.0429 (17)
N1	0.0728 (11)	0.0384 (8)	0.0437 (8)	−0.0004 (7)	−0.0091 (7)	0.0037 (6)
N2	0.0701 (11)	0.0454 (9)	0.0487 (8)	0.0024 (7)	−0.0149 (8)	0.0007 (7)
N3	0.0583 (11)	0.0499 (9)	0.0610 (10)	0.0037 (8)	−0.0101 (8)	0.0004 (7)
N4	0.0679 (11)	0.0538 (10)	0.0491 (9)	0.0028 (8)	−0.0096 (8)	0.0018 (7)
O1	0.0996 (11)	0.0519 (8)	0.0645 (8)	0.0159 (8)	−0.0274 (8)	−0.0014 (7)
O2	0.0886 (11)	0.0569 (9)	0.0785 (10)	0.0038 (7)	−0.0264 (8)	0.0104 (7)

### Geometric parameters (Å, °)

**Table:**

C1—C6	1.369 (3)	C19—C25	1.506 (2)
C1—C2	1.373 (3)	C20—C21	1.369 (3)
C1—C7	1.513 (2)	C20—H20	0.9300
C2—C3	1.379 (3)	C21—C22	1.355 (4)
C2—H2	0.9300	C21—H21	0.9300
C3—C4	1.349 (3)	C22—C23	1.350 (3)
C3—H3	0.9300	C22—H22	0.9300
C4—C5	1.364 (3)	C23—C24	1.369 (3)
C4—H4	0.9300	C23—H23	0.9300
C5—C6	1.381 (3)	C24—H24	0.9300
C5—H5	0.9300	C25—N3	1.459 (2)
C6—H6	0.9300	C25—C29	1.527 (3)
C7—N1	1.455 (2)	C25—H25	0.9800
C7—C11	1.535 (2)	C26—N3	1.465 (2)
C7—H7	0.9800	C26—C31	1.496 (3)
C8—N1	1.462 (2)	C26—C27	1.539 (3)
C8—C13	1.511 (2)	C26—H26	0.9800
C8—C9	1.539 (2)	C27—N4	1.469 (2)
C8—H8	0.9800	C27—C30	1.514 (3)
C9—N2	1.463 (2)	C27—H27	0.9800
C9—C12	1.511 (2)	C28—O2	1.234 (2)
C9—H9	0.9800	C28—N4	1.321 (2)
C10—O1	1.236 (2)	C28—C29	1.492 (3)
C10—N2	1.322 (2)	C29—H29A	0.9700
C10—C11	1.493 (2)	C29—H29B	0.9700
C11—H11A	0.9700	C30—H30A	0.9600
C11—H11B	0.9700	C30—H30B	0.9600
C12—H12A	0.9600	C30—H30C	0.9600
C12—H12B	0.9600	C31—C36	1.377 (3)
C12—H12C	0.9600	C31—C32	1.378 (4)
C13—C14	1.377 (3)	C32—C33	1.382 (4)
C13—C18	1.381 (2)	C32—H32	0.9300
C14—C15	1.385 (3)	C33—C34	1.373 (7)
C14—H14	0.9300	C33—H33	0.9300
C15—C16	1.369 (3)	C34—C35	1.363 (8)
C15—H15	0.9300	C34—H34	0.9300
C16—C17	1.365 (3)	C35—C36	1.366 (5)
C16—H16	0.9300	C35—H35	0.9300
C17—C18	1.376 (3)	C36—H36	0.9300
C17—H17	0.9300	N1—H1A	0.876 (19)
C18—H18	0.9300	N2—H2A	0.90 (2)
C19—C24	1.364 (3)	N3—H3A	0.87 (2)
C19—C20	1.372 (3)	N4—H4A	0.87 (2)
			
C6—C1—C2	117.44 (18)	C22—C21—C20	120.8 (2)
C6—C1—C7	123.39 (17)	C22—C21—H21	119.6
C2—C1—C7	119.14 (17)	C20—C21—H21	119.6
C1—C2—C3	121.5 (2)	C23—C22—C21	119.0 (2)
C1—C2—H2	119.2	C23—C22—H22	120.5
C3—C2—H2	119.2	C21—C22—H22	120.5
C4—C3—C2	120.2 (2)	C22—C23—C24	120.5 (2)
C4—C3—H3	119.9	C22—C23—H23	119.7
C2—C3—H3	119.9	C24—C23—H23	119.7
C3—C4—C5	119.4 (2)	C19—C24—C23	121.3 (2)
C3—C4—H4	120.3	C19—C24—H24	119.3
C5—C4—H4	120.3	C23—C24—H24	119.3
C4—C5—C6	120.5 (2)	N3—C25—C19	110.16 (14)
C4—C5—H5	119.8	N3—C25—C29	110.58 (16)
C6—C5—H5	119.8	C19—C25—C29	108.39 (15)
C1—C6—C5	120.9 (2)	N3—C25—H25	109.2
C1—C6—H6	119.5	C19—C25—H25	109.2
C5—C6—H6	119.5	C29—C25—H25	109.2
N1—C7—C1	110.20 (14)	N3—C26—C31	108.24 (16)
N1—C7—C11	111.32 (14)	N3—C26—C27	111.45 (15)
C1—C7—C11	108.75 (14)	C31—C26—C27	110.15 (15)
N1—C7—H7	108.8	N3—C26—H26	109.0
C1—C7—H7	108.8	C31—C26—H26	109.0
C11—C7—H7	108.8	C27—C26—H26	109.0
N1—C8—C13	107.78 (13)	N4—C27—C30	106.14 (15)
N1—C8—C9	111.05 (14)	N4—C27—C26	112.67 (15)
C13—C8—C9	112.28 (14)	C30—C27—C26	112.83 (16)
N1—C8—H8	108.5	N4—C27—H27	108.3
C13—C8—H8	108.5	C30—C27—H27	108.3
C9—C8—H8	108.5	C26—C27—H27	108.3
N2—C9—C12	106.57 (14)	O2—C28—N4	121.56 (17)
N2—C9—C8	111.11 (14)	O2—C28—C29	120.27 (18)
C12—C9—C8	113.98 (15)	N4—C28—C29	118.17 (17)
N2—C9—H9	108.3	C28—C29—C25	115.22 (16)
C12—C9—H9	108.3	C28—C29—H29A	108.5
C8—C9—H9	108.3	C25—C29—H29A	108.5
O1—C10—N2	121.76 (17)	C28—C29—H29B	108.5
O1—C10—C11	120.52 (16)	C25—C29—H29B	108.5
N2—C10—C11	117.72 (16)	H29A—C29—H29B	107.5
C10—C11—C7	114.13 (15)	C27—C30—H30A	109.5
C10—C11—H11A	108.7	C27—C30—H30B	109.5
C7—C11—H11A	108.7	H30A—C30—H30B	109.5
C10—C11—H11B	108.7	C27—C30—H30C	109.5
C7—C11—H11B	108.7	H30A—C30—H30C	109.5
H11A—C11—H11B	107.6	H30B—C30—H30C	109.5
C9—C12—H12A	109.5	C36—C31—C32	118.7 (2)
C9—C12—H12B	109.5	C36—C31—C26	120.3 (2)
H12A—C12—H12B	109.5	C32—C31—C26	120.9 (2)
C9—C12—H12C	109.5	C31—C32—C33	120.8 (4)
H12A—C12—H12C	109.5	C31—C32—H32	119.6
H12B—C12—H12C	109.5	C33—C32—H32	119.6
C14—C13—C18	118.40 (16)	C34—C33—C32	119.5 (5)
C14—C13—C8	121.63 (16)	C34—C33—H33	120.2
C18—C13—C8	119.96 (16)	C32—C33—H33	120.2
C13—C14—C15	120.50 (18)	C35—C34—C33	119.5 (4)
C13—C14—H14	119.8	C35—C34—H34	120.2
C15—C14—H14	119.8	C33—C34—H34	120.2
C16—C15—C14	120.38 (19)	C34—C35—C36	121.2 (5)
C16—C15—H15	119.8	C34—C35—H35	119.4
C14—C15—H15	119.8	C36—C35—H35	119.4
C17—C16—C15	119.41 (18)	C35—C36—C31	120.3 (4)
C17—C16—H16	120.3	C35—C36—H36	119.9
C15—C16—H16	120.3	C31—C36—H36	119.9
C16—C17—C18	120.56 (19)	C7—N1—C8	118.18 (14)
C16—C17—H17	119.7	C7—N1—H1A	107.0 (12)
C18—C17—H17	119.7	C8—N1—H1A	106.2 (12)
C17—C18—C13	120.75 (19)	C10—N2—C9	125.64 (16)
C17—C18—H18	119.6	C10—N2—H2A	117.7 (13)
C13—C18—H18	119.6	C9—N2—H2A	116.6 (13)
C24—C19—C20	117.58 (19)	C25—N3—C26	117.12 (15)
C24—C19—C25	121.33 (17)	C25—N3—H3A	107.6 (15)
C20—C19—C25	120.94 (18)	C26—N3—H3A	106.1 (15)
C21—C20—C19	120.7 (2)	C28—N4—C27	127.07 (16)
C21—C20—H20	119.6	C28—N4—H4A	117.6 (14)
C19—C20—H20	119.6	C27—N4—H4A	114.3 (14)
			
C6—C1—C2—C3	−1.7 (3)	C24—C19—C25—N3	−137.32 (19)
C7—C1—C2—C3	176.56 (19)	C20—C19—C25—N3	47.3 (3)
C1—C2—C3—C4	0.6 (4)	C24—C19—C25—C29	101.6 (2)
C2—C3—C4—C5	1.0 (4)	C20—C19—C25—C29	−73.8 (2)
C3—C4—C5—C6	−1.5 (4)	N3—C26—C27—N4	−75.63 (19)
C2—C1—C6—C5	1.2 (3)	C31—C26—C27—N4	164.21 (17)
C7—C1—C6—C5	−177.0 (2)	N3—C26—C27—C30	164.19 (16)
C4—C5—C6—C1	0.3 (4)	C31—C26—C27—C30	44.0 (2)
C6—C1—C7—N1	−14.8 (2)	O2—C28—C29—C25	119.9 (2)
C2—C1—C7—N1	167.09 (17)	N4—C28—C29—C25	−59.8 (2)
C6—C1—C7—C11	107.5 (2)	N3—C25—C29—C28	81.2 (2)
C2—C1—C7—C11	−70.6 (2)	C19—C25—C29—C28	−157.99 (16)
N1—C8—C9—N2	79.72 (18)	N3—C26—C31—C36	137.3 (2)
C13—C8—C9—N2	−159.53 (15)	C27—C26—C31—C36	−100.6 (2)
N1—C8—C9—C12	−159.86 (16)	N3—C26—C31—C32	−46.1 (3)
C13—C8—C9—C12	−39.1 (2)	C27—C26—C31—C32	75.9 (2)
O1—C10—C11—C7	−113.7 (2)	C36—C31—C32—C33	0.6 (4)
N2—C10—C11—C7	65.3 (2)	C26—C31—C32—C33	−176.0 (2)
N1—C7—C11—C10	−79.9 (2)	C31—C32—C33—C34	0.9 (5)
C1—C7—C11—C10	158.55 (16)	C32—C33—C34—C35	−2.1 (6)
N1—C8—C13—C14	40.2 (2)	C33—C34—C35—C36	1.9 (7)
C9—C8—C13—C14	−82.4 (2)	C34—C35—C36—C31	−0.4 (5)
N1—C8—C13—C18	−140.06 (17)	C32—C31—C36—C35	−0.9 (4)
C9—C8—C13—C18	97.33 (19)	C26—C31—C36—C35	175.7 (2)
C18—C13—C14—C15	−0.1 (3)	C1—C7—N1—C8	−173.45 (15)
C8—C13—C14—C15	179.61 (17)	C11—C7—N1—C8	65.8 (2)
C13—C14—C15—C16	0.5 (3)	C13—C8—N1—C7	169.15 (15)
C14—C15—C16—C17	−0.5 (3)	C9—C8—N1—C7	−67.5 (2)
C15—C16—C17—C18	0.3 (3)	O1—C10—N2—C9	−179.67 (18)
C16—C17—C18—C13	0.1 (3)	C11—C10—N2—C9	1.4 (3)
C14—C13—C18—C17	−0.1 (3)	C12—C9—N2—C10	168.85 (18)
C8—C13—C18—C17	−179.88 (17)	C8—C9—N2—C10	−66.4 (2)
C24—C19—C20—C21	0.0 (4)	C19—C25—N3—C26	169.44 (16)
C25—C19—C20—C21	175.5 (2)	C29—C25—N3—C26	−70.8 (2)
C19—C20—C21—C22	−1.3 (5)	C31—C26—N3—C25	−169.93 (17)
C20—C21—C22—C23	1.5 (5)	C27—C26—N3—C25	68.8 (2)
C21—C22—C23—C24	−0.4 (4)	O2—C28—N4—C27	174.06 (18)
C20—C19—C24—C23	1.2 (3)	C29—C28—N4—C27	−6.2 (3)
C25—C19—C24—C23	−174.4 (2)	C30—C27—N4—C28	−170.8 (2)
C22—C23—C24—C19	−1.0 (4)	C26—C27—N4—C28	65.3 (2)

### Hydrogen-bond geometry (Å, °)

**Table:**

*D*—H···*A*	*D*—H	H···*A*	*D*···*A*	*D*—H···*A*
C6—H6···N1	0.93	2.45	2.797 (2)	102
N2—H2A···O2^i^	0.90 (2)	2.02 (2)	2.911 (2)	170.2 (18)
N4—H4A···O1^i^	0.87 (2)	2.03 (2)	2.884 (2)	167 (2)
N1—H1A···Cg1^ii^	0.88 (2)	2.93 (2)	3.707 (2)	149.5 (2)
C18—H18···Cg5^iii^	0.93	2.95	3.872 (2)	171

Symmetry codes: (i) −*x*, −*y*, −*z*+1; (ii) −*x*, −*y*, −*z*; (iii) *x*+1, *y*, *z*.
